# Lung function discordance in monozygotic twins and associated differences in blood DNA methylation

**DOI:** 10.1186/s13148-017-0427-2

**Published:** 2017-12-21

**Authors:** Anneli C. S. Bolund, Anna Starnawska, Martin R. Miller, Vivi Schlünssen, Vibeke Backer, Anders D. Børglum, Kaare Christensen, Qihua Tan, Lene Christiansen, Torben Sigsgaard

**Affiliations:** 1 Department of Public Health, Section for Environment Occupation and Health, Danish Ramazzini Centre, Aarhus University, Aarhus, Denmark; 2 The Lundbeck Foundation Initiative for Integrative Psychiatric Research, iPSYCH, Aarhus, Denmark; 3 Department of Biomedicine, Aarhus University, Wilhelm Meyers Alle 4, 8000 Aarhus, Denmark; 4 Center for Integrative Sequencing, iSEQ, Aarhus University, Aarhus, Denmark; 5 Institute of Occupational and Environmental Medicine, University of Birmingham, Birmingham, UK; 6 National Research Centre for the Working Environment, Copenhagen, Denmark; 7 Department of Respiratory Medicine, Bispebjerg University Hospital, Copenhagen, Denmark; 8 The Danish Twin Registry, Institute of Public Health, University of Southern Denmark, Odense, Denmark; 9 The Danish Aging Research Center, Epidemiology, Biostatistics and Biodemography, Institute of Public Health, University of Southern Denmark, Odense, Denmark; 10 Department of Clinical Genetics, Odense University Hospital, Odense, Denmark; 11 Department of Clinical Biochemistry and Pharmacology, University Hospital, Odense, Denmark

**Keywords:** Lung function, Cross-sectional, Longitudinal, Blood, DNA methylation, EWAS, Epigenetics, Monozygotic twins, Epidemiology

## Abstract

**Background:**

Lung function is an important predictor of morbidity and mortality, with accelerated lung function decline reported to have immense consequences for the world’s healthcare systems. The lung function decline across individual’s lifetime is a consequence of age-related changes in lung anatomical structure and combination of various environmental factors; however, the exact molecular mechanisms contributing to this decline are not fully understood. DNA methylation is an epigenetic modification that changes across individual’s lifetime, as well as allows for interplay between environmental and genetic factors. DNA methylation plays a crucial role in regulation of gene expression, with increasing evidence linking aberrant DNA methylation levels with a number of common human diseases. In this study, we investigated possible associations between genome-wide DNA methylation levels and lung function in 169 pairs of middle-aged monozygotic twins (86 male pairs: mean age (min-max) = 66 years (57–79); 83 female pairs: mean age (min-max) = 66 years (56–78)). The twins were collected from the Danish Twin Registry and were examined at baseline (1998–1999) and follow-up (2008–2011) visits. Using the twin design, we correlated intra-pair differences in cross-sectional and longitudinal lung function with intra-pair blood DNA methylation differences at follow-up by linear regression analyses adjusted for sex, age, BMI, smoking, and blood cell composition measured for each individual with the use of flow cytometry.

**Results:**

We identified several differentially methylated CpG sites associated with forced expiratory volume the first second (FEV1) and forced vital capacity (FVC). Three probes identified for level of FVC were located in *GLIPR1L2* gene (lowest *p* value = 7.14 × 10−8), involved in innate immunity and tumour-suppressor/pro-oncogenic mechanisms. Change in FEV1 during the 11-year follow-up period was associated with blood DNA methylation level in *TRIM27* gene (*p* value = 1.55 × 10^−6^), a negative regulator of CD4 T cells, and also involved in cancer development. Several enriched pathways were identified, especially for FEV1, with one being “TGFBR” (Benjamini-Hochberg_adj_ *p* value = 0.045), the receptor for TGFβ, a growth factor involved in normal lung tissue repair through pro-fibrotic effects.

**Conclusions:**

Our findings suggest that epigenetic regulation of immunological- and cancer-related genes, as well as TGF-β-receptor-related genes, may be involved in the cross-sectional level and longitudinal change in lung function in middle-aged monozygotic twins.

**Electronic supplementary material:**

The online version of this article (10.1186/s13148-017-0427-2) contains supplementary material, which is available to authorized users.

## Background

Accelerated decline in lung function and associated diseases can have immense consequences for the individual and profound economic and social consequences for society [1, 2]. Lung function is an important predictor of morbidity and mortality [3], as well as cognitive and physical well-being in the general population [4]. Even though lung function slowly and continuously declines with age in adulthood [5], an accelerated decline in lung function is associated with chronic pulmonary diseases, such as asthma and chronic obstructive pulmonary disease (COPD). Both diseases impose a high burden on society; COPD is estimated to be the third leading cause of death worldwide [6], and the prevalence of asthma is increasing, now affecting more than 10% of the population in some developed countries [1].

Age-dependent lung function decline is a consequence of anatomical structural changes, such as decreased lung recoil and decreased respiratory muscle strength, in combination with physiological and immunological changes [7]. Furthermore, factors such as smoking [8] and various occupational exposures [9] are known to lead to an accelerated decline in lung function.

Studies on monozygotic (MZ) and dizygotic twins have previously shown a substantial genetic component influencing level of lung function, with heritability estimates for forced expiratory volume in the first second (FEV1) ranging from 61 to 69%, and for forced vital capacity (FVC) from 55 to 63% [10, 11]. Part of this heritability is linked to body composition, such as height and chest size [12]. Furthermore, several genome-wide association studies (GWAS) have identified numerous genetic variants associated with lung function level and change [13–15], as well as identified important integrative pathways for lung function and airflow obstruction [16, 17]. However, as environmental factors are also known to impact individual’s lung function, novel molecular mechanisms, such as epigenetics, may help understand the variance in liability and systems biology responsible for change in lung function across lifetime.

Epigenetics, literally meaning “beyond the genetics”, is the science of heritable changes in gene regulation and expression that do not involve changes to the underlying DNA sequence. Epigenetic modifications, such as DNA methylation and histone modifications, alter chromatin structure and DNA accessibility and thereby regulate patterns of gene expression in the genome [18–25]. The most widely studied epigenetic modification is DNA methylation, which primarily occurs at CpG sites [26]. Direction of correlation between DNA methylation and gene expression is dependent on the genomic context. Overall, DNA methylation in promoter regions and at repetitive elements is associated with decreased gene expression, in gene bodies with increased expression, while its presence in intergenic or 3′-untranslated regions is associated with upregulation of some transcripts and downregulation of others [19, 26–32]. Genome-wide DNA methylation patterns change along lifetime due to environmental, as well as stochastic factors impacting epigenome [33]. The dynamic nature of this epigenetic modification is possible due to two main groups of enzymes, DNA methyltransferases (DNMTs) and ten-eleven translocation (TET) proteins, responsible for addition, maintanance, and removal of methyl groups from DNA sequences at a pre-designed manner [34–39]. Improper functioning of this writing, reading, and erasing epigenetic machinery is associated with development and progression of multiple diseases, such as diabetes, cancer, and neurodegenerative and mental disorders [40–46]. Moreover, differences in DNA methylation levels between MZ discordant twins were associated before with several common disorders, including cancer, autoimmune diseases, diabetes, as well as mental disorders [47–51].

There are various environmental factors that may influence lung function and epigenetic modifications along lifetime, with smoking considered to be a strong environmental modifier of DNA methylation [52–54] and histone modifications [55–58], as well as the greatest predictor of lung function decline [8]. Several studies [52, 59] have found effects of smoking to be associated with both hypo- and hyper-methylation signatures depending on the CpG site, although global hypo-methylation seems to dominate in smokers [52, 60]. Only few studies have explored the association between DNA methylation and the cross-sectional level and longitudinal change in lung function. Significant associations have been found between methylation of the inflammatory genes *CRAT*, *F3*, *TLR2*, *IFNγ*, and *IL6*, as well as *SERPINA1*, *ATP6V1E2*, *FXYD1*, *FUT7*, *STAT5A*, *TRPM2*, and *LRP3*, and lung function [61, 62]. Also, the methylation of the repetitive elements *Alu* and LINE-1 was found to be associated with lung function [63].

In this study, we performed an epigenome-wide association study (EWAS) between lung function and genome-wide DNA methylation levels using a study sample of 169 middle-aged MZ twin pairs, thus enabling us to control for underlying genetic and shared environmental factors. We explored blood DNA methylation signatures in association with both cross-sectional lung function level and long-term change in lung function during an 11-year follow-up period.

## Methods

The studied population is a sub-population of twins from the middle-aged Danish twin (MADT) study [64] collected as a part of the Danish Twin Register (DTR) [65]. MADT was initiated with a baseline survey in 1998–1999 as a Danish nation-wide study of 4314 twins randomly selected from birth cohorts spanning 1931–1952 [64]. A follow-up study was conducted in 2008–2011 of all eligible twin pairs (9.9% deceased) originally enrolled [66]. The present study included 169 MZ twin pairs (83 female and 86 male pairs) that participated at both baseline and follow-up and with full data available.

Lung function was assessed for all participants at baseline at the participant’s home [10] and at the follow-up approximately 11 years later (min-max 9.6–13.4 years) at five study centres [66]. The three lung function measures FEV1, FVC, and the ratio FEV1/FVC (differentiating between obstructive and restrictive pulmonary disease) were assessed by spirometry using the micro DL device at baseline and “EasyOne” device at follow-up. The quality of each attempt was evaluated, and the highest obtained spirometry values out of three acceptable attempts for each individual were accepted and included for further analyses according to spirometry guidelines [67]. Height and weight was self-reported at baseline and measured at follow-up. Body mass index (BMI) was calculated at both time points as weight (kg) divided by height squared (m^2^). At follow-up, whole blood samples were collected from all participants. Informed written consent was obtained from all participants. Written informed consents obtained from all participants and the surveys, including collection of blood and use of survey information, were approved by the Regional Committees on Health Research Ethics for Southern Denmark (S-VF-19980072).

In order to standardize each participant’s individual lung function, the important predictors sex, age, height, and ethnicity, as well as the lung function measures of the individual, were applied to the GLI2012 equations [68] providing *z*-scores for FEV1, FVC, and FEV1/FVC for both baseline and follow-up measures. The standardized *z*-scores hence describe how many standard deviations (SDs) an individual’s lung function is away from the lung function of a healthy, non-smoking reference population of same sex, age, height, and ethnicity. We used follow-up height for both baseline and follow-up *z*-score calculations, as height was only objectively measured at the follow-up visit. Moreover, for this middle-aged group of twins, it was not expected that changes in height during the follow-up period would occur. Calculation of change in *z*-score during the follow-up period for all three lung function measures was done by subtracting the baseline *z*-score from the follow-up *z*-score.

Intra-pair (IP) differences in *z*-score for level of lung function (ΔzFEV1_IP_, ΔzFVC_IP_, ΔzFEV1/FVC_IP_) were calculated for follow-up values as the absolute difference in e.g. zFEV1 between the “superior” twin (the twin with higher zFEV1) and the “inferior” twin (the twin with lower zFEV1):

$$ \Delta \mathrm{zFEV}{1}_{\mathrm{IP}}=\mathrm{zFEV}{1}_{\mathrm{superior}}-\mathrm{zFEV}{1}_{\mathrm{inferior}} $$

Similarly, ΔzFVC_IP_ and ΔzFEV1/FVC_IP_ were calculated as the difference between the “superior” and the “inferior” twin, respectively. The same was calculated for intra-pair differences of the changes in lung function *z*-scores during the follow-up period (ΔzFEV1-change_IP_, ΔzFVC-change_IP_, ΔzFEV1/FVC-change_IP_).

All analyses were adjusted for smoking status and smoking history of the investigated twins. Smoking was expressed as pack years (equal to 20 cigarettes/day × years) in total and during the follow-up period. Participants were also divided into current smokers, non-current smokers, and never smokers at follow-up. Smokers who quit smoking less than 2 years prior to assessment were defined as current smokers at the time of assessment in order to account for smoking effects that persist for some time after smoking cessation.

### DNA methylation analysis

The semi-automated salt precipitation protocol with Autopure System (Qiagen) was used to extract genomic DNA from leukocytes in the buffy coat. Genomic DNA (500 ng/sample) was bisulfite-converted with EZ Methylation Gold Kit (Zymo Research) and analysed using the Infinium HumanMethylation450 BeadChips (Illumina) array according to the manufacturer’s protocol. Quality control for obtained DNA methylation data was performed with two different pipelines, a combination of MethylAid [69] and minfi tools [70]. Probes with low bead count (< 3 beads), high detection *p* value (> 0.01), and zero signal and missing in > 5% of samples were removed from further analysis. Additionally, cross-reactive probes identified previously by Chen et al. [71] were removed from the dataset. Four hundred fifty-three thousand fourteen good quality probes remained for further EWAS analyses. Normalisation of DNA methylation data, in order to control for technical variation, was done with the use of functional normalization (FunNorm) [72], and obtained *β* values (the proportion of DNA methylation) were further logit-transformed giving *M* values  for each probe.

### Blood cell composition

Blood cell counts were measured from the same blood samples that were used for DNA methylation profiling. Blood cell counts were available for 332 individuals, for which blood leukocyte subtypes (monocytes, lymphocytes, basophils, neutrophils, and eosinophils) were counted using a Coulter LH 750 Haematology Analyser. Blood cell counts were not available for six individuals, and thus, they were imputed based on the methylome dataset as described previously by van Iterson, pipeline provided on GitHub [73]. Blood cell counts were used to adjust for individual differences in cellular heterogeneity in blood sample from which genomic DNA was extracted.

### Statistical analyses

Distributions of data were evaluated using histograms and quantile-quantile plots. For normally distributed data, mean ± SD was reported, and comparisons were made using Student’s *t* test. For non-normally distributed data, median (min-max) was reported and a Mann-Whitney (Wilcoxon) rank-sum test was used to compare groups with unequal variance.

Epigenome-wide association study (EWAS) analyses were performed for intra-pair (IP) differences of both *cross-sectional* and *longitudinal* lung function *z*-scores.

The intra-pair differences in DNA methylation level (*M* value) for each probe was calculated as the “superior” minus the “inferior” twin in accord with the explanatory variable (e.g. ΔzFEV1_ip_). The same was done for all other included variables for each twin pair. In EWAS analyses, using linear regression models, associations between intra-pair DNA methylation difference and both the cross-sectional and the longitudinal intra-pair lung function difference were investigated. The *z*-score of lung function can be seen as a relative measure, as it implies the state of the individuals’ lung function, compared to the state expected from the reference population (GLI 2012 [68]). With that consideration and also due to the distribution of *z*-scores, which is around 0, we took the intra-pair difference in *z*-score as a measurement of quantitative discordance, instead of calculating the proportion of discordance between twins as done in previous studies of birth weight discordant MZ twins [74, 75].

All regression models were adjusted for sex, age, BMI, ever smoking history (total pack-years), and smoking status at follow-up, as well as blood cell composition difference within each twin pair. The longitudinal models were additionally adjusted for BMI change during the follow-up period (instead of cross-sectional BMI) and smoking pack-years during the follow-up period (instead of total pack-years).

Log transformation was applied to independent variables with extremely skewed distributions (IP differences of zFEV1, zFVC, zFEV1-change, and zFVC-change). All analyses were performed in *R* (http://www.R-project.org/) or STATA14 (StataCorp. 2015. Stata Statistical Software: Release 14. College Station, TX: StataCorp LP).

Results with a *p* value < 10^−6^ were reported as significant in this study (as suggested to be the genome-wide significant threshold for EWAS [76]). The level of significance for the corresponding false-discovery rate (FDR)-adjusted *p* value was < 0.05. Results from EWAS with a *p* value < 10^−5^ were presented in tables.

In order to explore if associated genes were overrepresented in specific pathways, pathway enrichment analyses were performed. Kyoto Encyclopaedia of Genes and Genomes (KEGG), Gene Ontology (GO) pathway, and Pathway Commons (PC) enrichment with the WEB-based GEne SeT AnaLysis Toolkit (WebGestalt) [77] were used against the genes included in the 450K DNA methylation array. Probes from EWAS with an unadjusted *p* value < 10^−5^ were used for pathway enrichment analyses. *p* values from pathway enrichment analyses were corrected for multiple testing with the Benjamini-Hochberg (BH) correction method [78].

## Results

A total of 169 twin pairs (83 female and 86 male pairs) with a mean age at follow-up of 66 years (min-max 56–79 years) were available with complete details on lung function measurements at baseline and follow-up, sex, age, height, smoking history, and DNA methylation data. Table 1 shows the demographics of the participants.

**Table 1:** Demographics of the cohort of MZ twin pairs included in this study

	Male (*n* = 172)	Female (*n* = 166)
Participating twin pairs	86	83
Age, years	66 (57–79)	66 (56–78)
Follow-up time, years	11.0 (9.6–13.2)	11.0 (9.8–13.4)
Height, cm	174.0 (158.8–194)*	161.9 (148.5–176.2)
Weight, kg	81.9 (56.2–118.8)*	67.7 (35–102.9)
BMI, kg/m^2^	27.0 (19.3–38.0)*	25.5 (13.8–38.1)
Smoking, pack-years (mean ± SD)	20.8 ± 26.9*	7.3 ± 12.8

Numbers are stated as median (min-max) unless otherwise specified

*p < 0.05 between male and female

In Table 2, the lung function values are presented as absolute lung function values, *z*-score values, and change in z-score during the follow-up period for the population. Furthermore, calculated intra-pair differences in z-scores at follow-up are given, as well as the intra-pair differences in change in *z*-score during the follow-up period. As expected, males and females differed in absolute values for FEV1 and FVC, whereas standardized measures of lung function (*z*-scores) showed no significant difference between the sexes. Absolute lung function measures declined during the follow-up period (Table 2). The observed decrease was slightly higher in males in comparison to females; however, it was not higher relative to the baseline value, for which both males and females decreased with 13% in FEV1 and 5% in FVC during the 11 years of follow-up (data not shown). The variance of intra-pair difference in lung function tended to be higher in male twin pairs in comparison to female twin pairs—possibly due to greater discordance in smoking history between male twins than between female twins (mean difference of 14 pack-years vs. 8 pack-years (*p* < 0.05), respectively, for smoking discordant twin pairs).

**Table 2:** Lung function values for the cohort with full data available shown as absolute values (litres (L)), *z*-scores and intra-pair differences

Lung function (mean ± SD)	Male (*n* = 172)	Female (*n* = 166)
FEV1 (L) follow-up	3.0 ± 0.7*	2.2 ± 0.5
FVC (L) follow-up	4.1 ± 0.9*	2.9 ± 0.6
FEV1/FVC follow-up	0.80 ± 0.09	0.82 ± 0.07
FEV1-change (L)	− 0.45 ± 0.4*	− 0.34 ± 0.2
FVC-change (L)	− 0.21 ± 0.6	− 0.18 ± 0.4
FEV1/FVC-change	0	0
zFEV1 follow-up	− 0.46 ± 1.33	− 0.42 ± 1.18
zFVC follow-up	− 0.23 ± 1.23	− 0.15 ± 1.04
zFEV1/FVC follow-up	− 0.47 ± 1.19	− 0.53 ± 0.97
zFEV1-change	0.03 ± 0.79	0.14 ± 0.73
zFVC-change	0.45 ± 0.92	0.57 ± 0.82
zFEV1/FVC-change	− 0.87 ± 1.18	− 0.86 ± 1.12
Intra-pair (IP) differences (median (min-max))	(86 pairs)	(83 pairs)
ΔzFEV1_IP_ follow-up	0.7 (0.01–5.3)	0.6 (0.002–2.8)
ΔzFVC_IP_ follow-up	0.6 (0.03–4.8)	0.6 (0.02–2.9)
ΔzFEV1/FVC_IP_ follow-up	0.7 (0.02–3.9)	0.5 (0.01–2.5)
ΔzFEV1-change_IP_	0.7 (0.005–5.2)	0.5 (0.02–2.9)
ΔzFVC-change_IP_	0.7 (0.005–4.7)	0.6 (0.04–3.0)
ΔzFEV1/FVC_IP_-change_IP_	1.1 (0.005–5.7)	1.0 (0.02–4.3)

*p < 0.05 between male and female

Tables 3 and 4 present results from EWAS analyses for intra-pair difference in level of lung function at follow-up and for intra-pair difference in change in lung function during the follow-up period, respectively. All probes with *p* value < 10^−5^ were annotated with the most proximal gene, genomic position, and CpG island context (according to Human Genome Issue hg19) and are presented together with regression estimates and *p* values.

**Table 3:** Overviw of results from EWAS analyses for intra-pair difference in level of lung function at follow-up (*p* value < 10^−5^)

Lung function measure	Probe	Estimate	*p* value	Chromosome	Bp (hg19)	Proximal gene	CGI feature	Methylation^a^ of “inferior” twin
Log-ΔzFEV1_IP_	cg04261072	− 0.054	4.33E−06	13	79,977,499	*RBM26*	Body-shelf	Hyper
	cg10196163	− 0.062	7.62E−06	14	10,556,0628	NA	IGR-shore	Hyper
	cg12552820	− 0.033	2.69E−06	1	2,231,925	*SKI*	Body-shore	Hyper
	cg13971574	− 0.037	9.65E−06	2	39,102,874	*MORN2*	TSS1500-island	Hyper
	cg18582260	− 0.079	3.99E−06	13	25,085,301	*PARP4*	5′UTR-shore	Hyper
	cg20552903	− 0.038	5.81E−06	6	33,289,678	*DAXX*	Body-shore	Hyper
	cg23759053	− 0.136	5.99E−06	7	34,173,999	*BMPER*	Body-open sea	Hyper
	cg23840275	− 0.045	2.62E−06	13	20,969,493	NA	IGR-shore	Hyper
	cg27180671	− 0.050	7.51E−06	17	65,527,566	*PITPNC1*	Body-open sea	Hyper
Log-ΔzFVC_IP_	cg00008488	− 0.0935	8.23E−06	5	175,199,915	NA	IGR-shore	Hyper
	cg00306721	− 0.0797	9.54E−06	11	62,477,480	*BSCL2*	TSS1500-island	Hyper
	cg01028379	0.0617	2.84E−06	7	4,798,471	*FOXK1*	Body-shore	Hypo
	cg02071292	− 0.2366	**7.14E−08** ^**FDR**^	12	75,785,097	*GLIPR1L2*	1stExon-island	Hyper
	cg07311024	− 0.1553	1.96E−06	12	75,785,089	*GLIPR1L2*	1stExon-island	Hyper
	cg15909232	− 0.1167	5.08E−06	7	156,235,420	NA	IGR-open sea	Hyper
	cg15942481	− 0.1366	8.77E−06	12	75,785,230	*GLIPR1L2*	Body-island	Hyper
	cg17154159	0.0832	6.02E−06	6	160,401,562	*IGF2R*	Body-open sea	Hypo
	cg22089890	− 0.0391	4.20E−06	17	48,708,077	NA	IGR – shelf	Hyper
	cg25249300	− 0.0660	**1.36E−07**	2	54,483,341	*TSPYL6*	1stExon – island	Hyper
ΔzFEV1/FVC_IP_	cg00347643	0.1747	7.88E−06	7	75,957,202	*YWHAG*	3′UTR-shore	Hypo
	cg00995220	0.1333	**2.13E−07**	6	56,259,582	NA	IGR-open sea	Hypo
	cg04697953	0.0849	4.43E−06	2	179,299,404	*PRKRA*	Body-open sea	Hypo
	cg06244016	0.1917	2.17E−06	6	151,186,511	*MTHFD1L*	TSS200-shore	Hypo
	cg07219303	0.1575	3.92E−06	4	100,140,905	*ADH6*	TSS1500-open sea	Hypo
	cg11980944	0.0863	8.55E−06	1	205,399,731	NA	IGR-open sea	Hypo
	cg13107302	0.0995	5.69E−06	17	75,237,970	NA	IGR-open sea	Hypo
	cg13912599	0.1229	2.66E−06	1	150,959,380	*ANXA9*	Body-open sea	Hypo
	cg18221862	− 0.1038	4.81E−06	2	193,059,230	*TMEFF2*	1stExon-island	Hyper
	cg18537205	− 0.1205	9.86E−06	10	114,575,091	*VTI1A*	Body-open sea	Hyper
	cg19529957	0.1367	5.50E−06	7	4,198,590	*SDK1*	Body-open sea	Hypo

Bold indicates significance *p* < 1 × 10^−6^. Probes were annotated with the most proximal gene, genomic position Bp (hg19), and CpG island context (CGI feature)

*Estimate* intra-pair difference in *M* value (logit-transformed beta), *FDR* false discovery rate < 0.05, *TSS200* region spanning from transcription start site (TSS) to 200 bp upstream of TSS, *TSS1500* region spanning from − 200 to − 1500 bp upstream of TSS, *5′UTR* 5′ untranslated region, *IGR* intergenic region, *NA* not applicable, *Hyper* hyper-methylation, *Hypo* hypo-methylation

^a^Relative DNA methylation of “inferior” twin compared to “superior” twin

**Table 4:** Overview of results from EWAS analyses for intra-pair difference in change in lung function during the follow-up period (*p* value < 10^−5^)

Lung function measure	Probe	Estimate	*p* value	Chromosome	Bp (hg19)	Proximal gene	CGI feature	Methylation^a^ of “inferior” twin
Log-ΔzFEV1-change_IP_	cg19484381	− 0.055	1.55E−06	6	28,890,673	*TRIM27*	Body-shore	Hyper
	cg27261494	− 0.081	5.77E−06	6	74,104,097	*DDX43*	TSS200-shore	Hyper
Log-ΔzFVC-change_IP_	cg12796186	− 0.066	3.28E−06	1	10,458,599	*PGD*	TSS1500-island	Hyper
	cg14514174	− 0.044	6.39E−06	9	99,181,512	*ZNF367*	TSS1500-island	Hyper
ΔzFEV1/FVC-change_IP_	cg00552805	0.0551	9.84E−06	7	44,119,858	*POLM*	Body-shore	Hypo
	cg06375580	0.0449	9.25E−06	12	42,538,820	*GXYLT1*	TSS200-island	Hypo
	cg12733656	0.0396	8.82E−06	7	6,388,695	*C7orf70*	TSS200-island	Hypo

Bold indicates significance *p* < 1 × 10^−6^. Probes were annotated with the most proximal gene, genomic position Bp (hg19), and CpG island context (CGI feature)

*Estimate* intra-pair difference in *M* value (logit-transformed beta), *TSS200* region spanning from transcription start site (TSS) to 200 bp upstream of TSS, *TSS1500* region spanning from − 200 to − 1500 bp upstream of TSS, *5′UTR* 5′ untranslated region, *IGR* intergenic region, *NA* not applicable, *hyper* hyper-methylation, *hypo* hypo-methylation

^a^Relative DNA methylation of “inferior” twin compared to “superior” twin

**Fig. 1:**
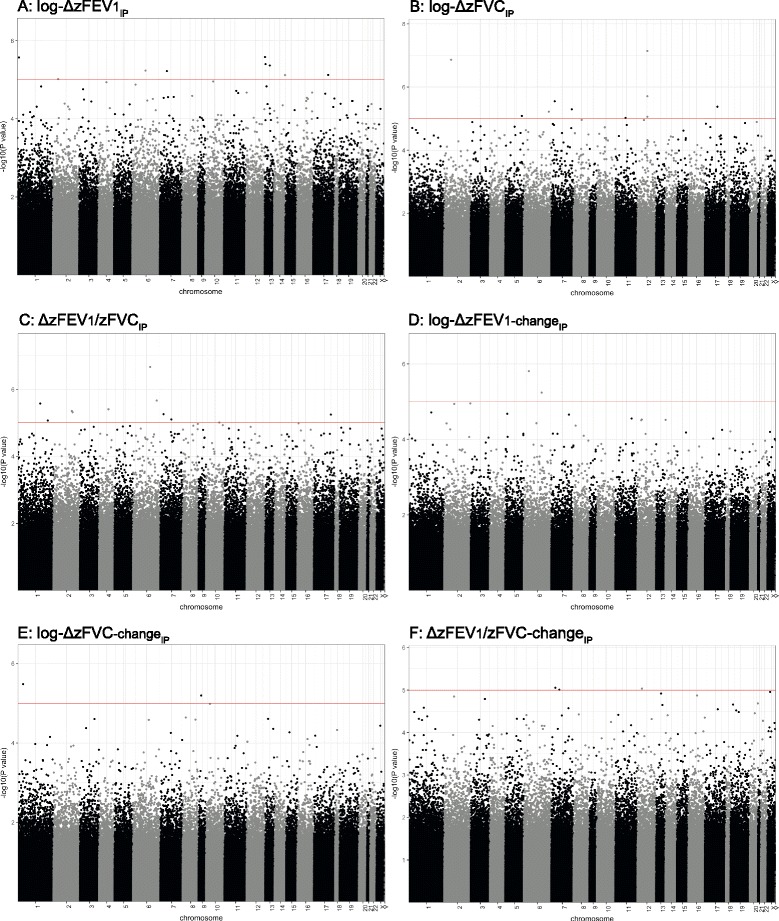
Manhattan plots depicting results from all six EWAS analyses

Manhattan plots depicting results from all six EWAS analyses are presented in Fig. 1a-f. In Additional file 1: Fig. S1A-F, the corresponding Q-Q plots are presented.

In regressing intra-pair DNA methylation differences on intra-pair differences in lung function at follow-up, nine CpG sites with a *p* value < 10^−5^ were found for zFEV1 (log-ΔzFEV1_IP_) (Table 3) with no sites reaching a *p* value < 10^−6^. For intra-pair difference in zFVC (log-ΔzFVC_IP_), ten sites with a *p* value < 10^−5^ were identified (Table 3), with the most significant probe identified as cg02071292 annotated to GLI (glioma) pathogenesis-related 1-like 2 (*GLIPR1L2*) with a *p* value = 7.14 × 10^−8^ (FDR_adj_ *p* value = 0.03). Two other probes with a *p* value < 10^−5^ in this analysis were also annotated to *GLIPR1L2* (cg07311024 *p* value = 1.96 × 10^−6^ and cg15942481 *p* value = 8.77 × 10^−6^). Intra-pair difference in level of zFEV1/FVC (log-ΔzFEV1/FVC_IP_) identified 11 CpG sites with the only probe reaching a *p* value < 10^−6^ mapping to no currently known gene (cg00995220, located on chromosome 6) (Table 3).

Regressing intra-pair DNA methylation differences on change in zFEV1 (log-ΔzFEV1-change_IP_) resulted in two findings of *p* value < 10^−5^ (Table 4), with the most associated probe cg19484381 mapping to tripartite motif containing 27 (*TRIM27*) with a *p* value = 1.55 × 10^−6^. Intra-pair difference for change in zFVC (log-ΔzFVC-change_IP_) also identified two associated probes, with the most highly associated probe cg12796186 mapping to phosphogluconate dehydrogenase (*PGD*) with a *p* value = 3.28 × 10^−6^. Change in zFEV1/FVC (ΔzFEV1/FVC-change_IP_) identified differential methylation at three probes (Table 4).

In general, the associated findings in both cross-sectional and longitudinal models showed higher DNA methylation (relative hyper-methylation) for zFEV1 and zFVC for the “inferior” twin, whereas lower DNA methylation (relative hypo-methylation) for zFEV1/FVC was seen for the “inferior” twin in identified probes (Tables 3 and 4).

Pathway enrichment analyses for GO, KEGG, and PC were performed on all EWAS results with a *p* value < 10^−5^ obtained from both cross-sectional and longitudinal lung function analyses, and the results are presented in Tables 5 and 6, respectively.

**Table 5:** Pathway enrichment analyses for Gene Ontology (GO), KEGG, and Pathway Commons (PC) (separate pathways with BH-corrected *p* value < 0.1) based on significant findings from EWAS for level of lung function

Lung function measure	Database	Pathway name	No. of genes	Genes	Statistics
Log-ΔzFEV1_IP_	GO	Negative regulation of BMP signaling pathway	2	*BMPER SKI*	*C* = 32; *O* = 2; *E* = 0.01; *R* = 142.86; rawP = 7.87e-05; **adjP = 0.0114**
		Ubiquitin protein ligase binding	2	*DAXX SKI*	*C* = 147; *O* = 2; *E* = 0.05; *R* = 38.69; rawP = 0.0010; **adjP = 0.0090**
		PML body	2	*DAXX SKI*	*C* = 72; *O* = 2; *E* = 0.02; *R* = 87.37; rawP = 0.0002; **adjP = 0.0052**
	PC	TGFBR	2	*DAXX SKI*	*C* = 125; *O* = 2; *E* = 0.05; *R* = 43.92; rawP = 0.0009; **adjP = 0.0450**
Log-ΔzFVC_IP_	No significant pathway enrichment results				
ΔzFEV1/FVC_IP_	GO	Protein homodimerization activity	3	*MTHFD1L ANXA9 PRKRA*	*C* = 554; *O* = 3; *E* = 0.27; *R* = 11.00; rawP = 0.0018; **adjP = 0.0324**
	KEGG	Metabolic pathways	2	*ADH6 MTHFD1L*	*C* = 1093; *O* = 2; *E* = 0.46; *R* = 4.39; rawP = 0.0720; adjP = 0.0720

Bold indicates BH adjP < 0.05

*C* the number of reference genes in the category, *O* the number of genes in the gene set and also in the category, *E* the expected number in the category, *R* ratio of enrichment, *rawP p* value from hypergeometric test, *adjP p* value adjusted by the multiple test adjustment (BH)

**Table 6:** Pathway enrichment analyses for Gene Ontology (GO), KEGG, and Pathway Commons (PC) (separate pathways with BH-corrected *p* value < 0.1) based on significant findings from EWAS for change in lung function

Lung function measure	Database	Pathway name	No. of genes	Genes	Statistics
Log-ΔzFEV1-change_IP_	No significant pathway enrichment results				
Log-ΔzFVC-change_IP_	No significant pathway enrichment results				
ΔzFEV1/FVC-change_IP_	GO	Transferase activity	2	*GXYLT1 POLM*	*C* = 1641; *O* = 2; *E* = 0.23; *R* = 8.67; rawP = 0.0133; **adjP = 0.0399**

Bold indicates BH adjP < 0.05

*C* the number of reference genes in the category; O: the number of genes in the gene set and also in the category, *E* the expected number in the category, *R* ratio of enrichment, *rawP p* value from hypergeometric test, *adjP p* value adjusted by the multiple test adjustment (BH)

Pathway enrichment analyses for GO, based on top results from intra-pair difference in level of zFEV1, identified several enriched pathways (Table 5). The most significant were “Negative regulation of bone morphogenetic protein (*BMP*) signalling pathway” (BH_adj_ *p* value = 0.011), “Ubiquitin protein ligase binding” (BH_adj_ *p* value = 0.009), and “Promyelocytic leukaemia protein (PML) body” (BH_adj_ *p* value = 0.005). Pathway Commons identified “Transforming growth factor beta (*TGF*-β) receptor (TGFBR)” to be an enriched pathway for zFEV1 (BH_adj_ *p* value = 0.045) (Table 5). Several other pathways driven by the same genes reached statistical significance (12 pathways with BH_adj_ *p* value < 0.05) (shown in Additional file 2: Table S1). Enrichment signal in all pathways for zFEV1 was driven by *SKI* (“Sloan-Kettering Institute”) proto-oncogene in combination with either BMP binding endothelial regulator (*BMPER*) or death domain-associated protein (*DAXX*). GO and KEGG pathway analyses for intra-pair difference in level of zFEV1/FVC identified enriched pathways to be “Protein homodimerization activity” and “Metabolic pathways” involving other genes than those highlighted for zFEV1 and zFVC (Table 5). Additional pathways found in all enrichment analyses are shown in Additional file 2: Table S1.

For intra-pair difference in change in lung function, pathway enrichment analyses identified significant pathways for ΔzFEV1/FVC-change_IP_ using Gene Ontology (Table 6). “Transferase activity” was identified as the most enriched pathway, driven by the gene xylosyltransferase 1 (*GXYLT1*) and DNA polymerase mu (*POLM*).

## Discussion

In this study, we investigated possible associations between lung function and genome-wide DNA methylation patterns in a population of middle-aged Danish MZ twin pairs. The lung function of the MADT cohort used in this study was assessed at two different occasions, which allowed us to investigate lung function both as cross-sectional level and as longitudinal change.

The EWAS analyses identified DNA methylation at several CpG sites associated with intra-pair difference in level of lung function for the different metrics. The most significant associated finding for the level of zFVC was DNA methylation of three CpG sites positioned in the gene body of *GLIPR1L2* in close proximity to each other (within 150 bp). This gene, situated on chromosome 12, is a member of the cysteine-rich secretory proteins, antigen 5, and pathogenesis-related 1 proteins (CAP) superfamily. These genes are expressed in the immune tissues and are involved in a variety of physiological processes, including innate immunity, inhibition of ion channels and proteases, and interaction with immunoglobulin proteins, as well as tumour suppressor and pro-oncogenic genes in different tissues [79, 80]. The *GLIPR1L2* is a p53 target gene encoding functional p53 response elements that induce tumour suppression [80]. p53 is the most widely and best described tumour suppressor gene known in humans, and p53 mutation is the most frequently described intermediate step in the path between smoking and lung cancer [81]. Additionally, as in this study we did not find any overlap with previous findings of differentially methylated loci associated with smoking [53], we feel confident that the adjustment for smoking status in these EWAS analyses was successful. However, apart from smoking status of an individual, other lifestyle choices (such as levels of physical activity) and environmental factors (such as pollution levels) impact both lung function levels and epigenetic modifications [5, 54–56, 82–86]. In the future, collection of detailed data on environmental factors and lifestyle choices of investigated individuals may help us identify epigenetic variation that mediates the effect of these exposures on lung function in a general population.

For intra-pair difference in change in lung function during the follow-up period, the EWAS analyses identified only few CpG sites to be associated with the studied trait. These were, among others, annotated to genes encoding cellular enzymes, i.e. *PGD*, *GXYLT1*, *POLM*, and DEAD box polypeptide 43 (*DDX43*), also known as *HAGE* (DEAD box helicase antigen), of which the latter has been shown to be an immunogenic cancer-specific antigen present in the protein level of different tumours including lung cancers [87]. Also, the methylation of *TRIM27*, a negative regulator of CD4 T cells [88] also involved in the development of cancer [89], was identified to be associated with change in lung function over the 11-year period.

The observed discrepancy in directions of level of DNA methylation within twin pairs (hypo-/hyper-methylation) for the different lung function measures (Tables 3 and 4) may illustrate differences in level of activation of inflammatory genes and pathways, especially for zFEV1/FVC, for which inflammation is known to be an important factor.

Pathway enrichment analyses showed several significant pathways of interest for the level of lung function. “Negative regulation of BMP signalling pathway” involving the genes *BMPER* and *SKI* and “TGFBR” pathway involving *DAXX* and *SKI* were enriched pathways for intra-pair difference in level of zFEV1. These pathways are involved in malignant tumour growth and metastasis [90, 91] and angiogenesis [92], and TGF-β plays an important role for normal lung morphogenesis and hence lung function [93]. TGF-β is involved in normal lung tissue repair in adults through its pro-fibrotic effects; however, over-expression of TGF-β is associated with different lung diseases, including lung fibrosis [93, 94]. Another possible mechanism for regulating the activity of TGF-β is the expression of the TGF-β receptors [93], making it plausible that the TGFBR pathway is of high importance for lung function. These findings suggest that DNA methylation of oncogenic- and tumour suppressor-related genes, as well as TGF-β-receptor-related genes, could be involved in the level and change in lung function.

Other studies similarly reported significant association between DNA methylation and lung function, however, not within the same genes and not involving the same pathways as found in this study, though most of the genes studied previously are included in the 450 BeadChips used in this study. Lepeule et al. [61] explored the association between the DNA methylation of nine specific inflammatory genes and the lung function level in a cohort of elderly Caucasian men. Decreased DNA methylation in the inflammatory genes *CRAT*, *F3*, and *TLR2* was shown to be associated with lower level of lung function (FEV1 and FVC). Oppositely, decreased DNA methylation in *IFNγ* and *IL6* was associated with better lung function, insinuating that these genes may be involved in activation of negative feedback in the inflammatory pathways [61]. Qiu et al. [62] identified 349 CpG sites significantly associated to lung function, of which 330 were hypo-methylated in the presence of lower lung function. Hypo-methylation of CpG sites in *SERPINA1* was negatively associated to both FEV1 and FEV1/FVC, and this was replicated in another cohort. This gene encodes the acute-phase reactant ɑ1-antitrypsin, a potent circulating anti-elastase produced in the liver but transported to the lung. Variation in the ɑ1-antitrypsin gene *SERPINA1* is known to be a monogenic cause of COPD [95], as deficiency of ɑ1-antitrypsin leads to failing maintenance of the structural integrity of the lung. Other top associations for CpG sites found by Qiu et al. were in *ATP6V1E2*, *FXYD1*, *FUT7*, and *STAT5A* for FEV1 and in *ATP6V1E2*, *FXYD1*, *TRPM2*, and *LRP3* for the FEV1/FVC ratio [62] with no overlap to our study. The specific associations between DNA methylation of the repetitive elements *Alu* and LINE-1 and lung function was studied in the Veterans Administration Normative Aging Study of older Caucasian men [63]. Hypo-methylation of *Alu* and LINE-1 was associated to lower cross-sectional FEV1 level. Faster rate of decline in FEV1 and FVC was associated with relative hypo-methylation in LINE-1 (*p* < 0.005), but not in *Alu*. The mechanism behind this association was suggested to be that hypo-methylation may increase the activity of the repetitive elements as retro-transposable sequences, leading to greater genomic instability and more mutations, which in turn may lead to adverse effects on lung function and lung function decline [63]. However, as the above-mentioned previous studies were performed on a general population and not on twin samples, their results may be driven by an underlying genetic component. Our study, comparing intra-pair methylation differences in MZ twins, accounts for the genetic factor for lung function and for DNA methylation levels.

It should be noted that blood samples from the participants in this study were collected only at follow-up. Accordingly, it was only possible to correlate longitudinal lung function change with DNA methylation status at follow-up and not with DNA methylation changes over time. Thus, although significant associations have been found between DNA methylation and lung function, the direction of causality cannot be inferred. However, biologically plausible hypotheses may suggest the direction of causality. The possible mechanism behind a causal relation may be that environmental exposures induce oxidative damage and changes in DNA methylation, which may in turn impact lung function due to altered gene expression, as also suggested by Lepeule et al. [63]. Additionally, as there is an increasing evidence suggesting association between smoking, histone modifications (e.g. histone acetylation), and lung function, future investigation of DNA methylation alongside with other epigenetic modifications and epigenome-modifying proteins (such as DNMTs, TETs, histone acetylases, and deacetylases) is warranted in order to reach deeper understanding of the role of epigenetics in lung function.

A possible limitation of this study is that DNA methylation was measured in leukocytes, as blood is an easily accessible biological sample. In this study, we adjusted all our analyses for blood cell counts measured with the use of flow cytometry for all individuals, and thus, we believe that our results are not cofounded by the blood cellular heterogeneity. Whether or not the identified methylation differences reflect differential processes also occurring in the lung tissue is not clear. However, leukocytes infiltrate the lung tissue and neutrophil inflammation can be an early component of lung function decline [61]. Furthermore, inflammatory cytokines have been shown to be elevated in circulating blood of COPD patients [96], suggesting a “spill-over” of cytokines from the lung tissue to the systemic circulation. This may also happen as part of the recruitment of leukocytes to the lungs in association with different inhalable exposures [97, 98]. DNA methylation levels of genes expressed in leukocytes may therefore be a relevant marker of e.g. inflammatory processes in the lungs. Chronic pulmonary diseases, such as asthma and COPD, are among the ten top leading causes of years’ lost due to disability worldwide and are associated with decreased lung function and accelerated lung function decline [99–103]. Due to the high prevalence of these diseases in a population, it is possible that the measurement of lung function in our investigated population reflected COPD or asthma diagnosis and thus that the differentially methylated sites identify loci associated with chronic pulmonary diseases. In the future, collection of detailed information on diagnosis and trajectory of chronic pulmonary diseases, alongside with FEV1 and FVC measures for the studied population, may help us delineate if the identified differentially methylated loci are also associated with chronic pulmonary diseases.

The observed cross-sectional lung function phenotype (*z*-score level) for this cohort was lower than expected for the population, while the z-score increased during the follow-up period in comparison to the GLI 2012 reference population. There might be several reasons for these cross-sectional and longitudinal differences. Firstly, different spirometer devices were used, and secondly, the increase in z-score during the follow-up period may be due to discrepancies when comparing longitudinal lung function change with that estimated from cross-sectional lung function prediction equations, possibly due to cohort effects as reported before [104]. However, we expect any possible misclassifications to be of non-differential type. The rate of absolute lung function decline for this age group was similar to what has been reported in previous studies [82, 83, 105].

As for generalizability to other populations, it must be emphasized that this unique cohort consisted of middle-aged Caucasian MZ twins with the unique opportunity to control for underlying genetic background, shared environment, and blood cell composition effects. How DNA methylation in younger cohorts and of other ethnicities would be associated with lung function still remains to be explored. Validation is further needed in order to ensure strength and relevance of our results.

## Conclusions

In conclusion, this study shows that DNA methylation patterns in blood are associated with the level and change in lung function in MZ twin pairs, identifying several CpG sites and biological pathways of possible importance for lung function. Specifically, oncogenic- and tumor suppressor-related genes (*GLIPR1L2*, *BMPER*, *SKI*, and *DAXX*), as well as TGF-β-receptor-related genes, could be involved in cross-sectional level and longitudinal change in lung function. All these findings point to biological pathways of potential importance for pulmonary physiology.

## Additional files

Additional file 1: **Figure S1(A-F).** (JPG 43 kb)

Additional file 2: Table S1. Additional pathways from enrichment analyses for Gene Ontology (GO), KEGG and Pathway Commons (PC) (BH-corrected *p*-value < 0.1) based on significant findings from EWAS for level of lung function. (DOCX 21 kb)

## Abbreviations

BH_adj_: Benjamini-Hochberg adjusted
BMI: Body mass index
COPD: Chronic obstructive pulmonary disease
CpG: Cytosine linked with a phosphodiester bond to guanine
DNA: Deoxyribonucleic acid
EWAS: Epigenome-wide association study
FEV1: Forced expiratory volume the first second
FVC: Forced vital capacity
GO: Gene ontology
IP: Intra-pair
MADT: Middle-aged Danish twins
MZ: Monozygotic
