# Supplementary figures and images for: Lung function discordance in monozygotic twins and associated differences in blood DNA methylation
Source: Clin Epigenetics. 2017 Dec 21;9:132. doi: 10.1186/s13148-017-0427-2 (PMC5740718; doi:10.1186/s13148-017-0427-2)

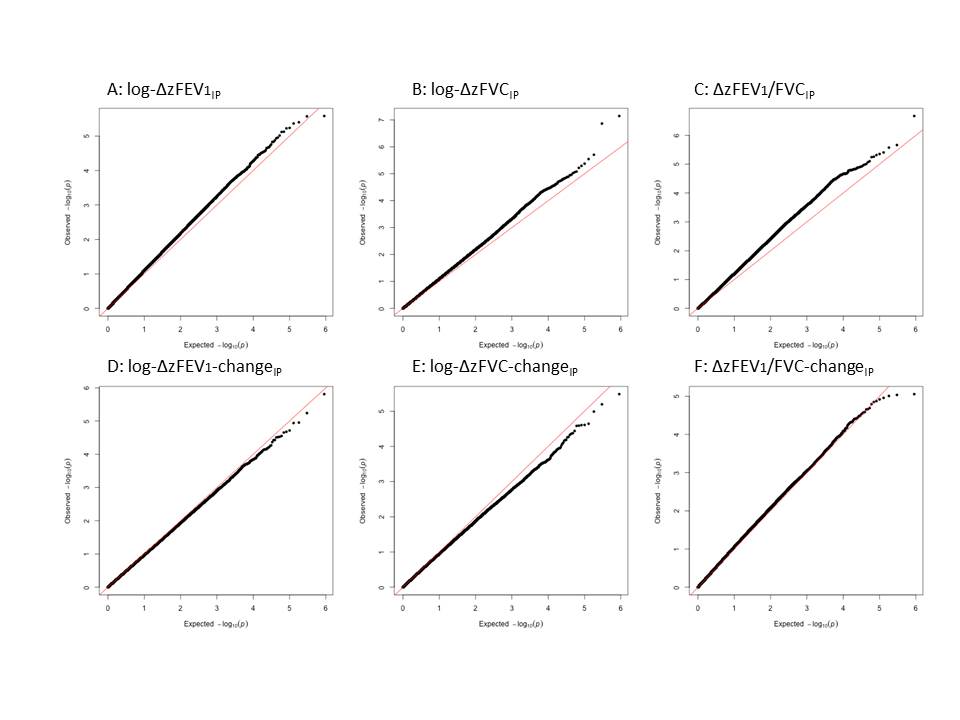

Supplement: Supplementary file 1 — Figure S1(A-F). (JPG 43 kb) [file 13148_2017_427_MOESM1_ESM.jpg]
