# Supplementary material for: Lung function discordance in monozygotic twins and associated differences in blood DNA methylation
Source: Clin Epigenetics. 2017 Dec 21;9:132. doi: 10.1186/s13148-017-0427-2 (PMC5740718; doi:10.1186/s13148-017-0427-2)
Supplement: Supplementary file 2 — Additional pathways from enrichment analyses for Gene Ontology (GO), KEGG and Pathway Commons (PC) (BH-corrected p-value < 0.1) based on significant findings from EWAS for level of lung function. (DOCX 21 kb) [file 13148_2017_427_MOESM2_ESM.docx]

**Table S1 Additional pathways from enrichment analyses for Gene Ontology (GO), KEGG and Pathway Commons (PC)**

**(BH-corrected p-value < 0.1) based on significant findings from EWAS for level of lung function**

| **Lung function measure** | **Data base** | **Pathway name** | **#Genes** | **Genes** | | **Statistics** | |
| --- | --- | --- | --- | --- | --- | --- | --- |
| **Log-ΔzFEV1_IP_** | GO | Regulation of BMP signaling pathway | 2 | *BMPER SKI* | | C=65; O=2; E=0.03; R=70.33; rawP=0.0003; adjP=0.0217 | |
|  |  | Negative regulation of transmembrane receptor protein serine/threonine kinase signaling pathway | 2 | *BMPER SKI* | | C=91; O=2; E=0.04; R=50.24; rawP=0.0006; adjP=0.0290 | |
|  |  | BMP signaling pathway | 2 | *BMPER SKI* | | C=106; O=2; E=0.05; R=43.13; rawP=0.0009; adjP=0.0326 | |
|  |  | Regulation of transmembrane receptor protein serine/threonine kinase signaling pathway | 2 | *BMPER SKI* | | C=163; O=2; E=0.07; R=28.05; rawP=0.0020; adjP=0.0580 | |
|  |  | Small conjugating protein ligase binding | 2 | *DAXX SKI* | | C=147; O=2; E=0.05; R=38.69; rawP=0.0010; adjP=0.0090 | |
|  |  | Enzyme binding | 3 | *DAXX SKI PARP4* | | C=1052; O=3; E=0.37; R=8.11; rawP=0.0036; adjP=0.0216 | |
|  |  | Transcription factor binding | 2 | *DAXX SKI* | | C=410; O=2; E=0.14; R=13.87; rawP=0.0078; adjP=0.0351 | |
|  |  | Nuclear body | 2 | *DAXX SKI* | | C=258; O=2; E=0.08; R=24.38; rawP=0.0026; adjP=0.0338 | |
|  | PC | ALK1 pathway | 2 | *DAXX SKI* | | C=320; O=2; E=0.12; R=17.15; rawP=0.0055; adjP=0.0458 | |
|  |  | Regulation of cytoplasmic and nuclear SMAD2/3 signaling | 2 | *DAXX SKI* | | C=301; O=2; E=0.11; R=18.24; rawP=0.0049; adjP=0.0458 | |
|  |  | Regulation of nuclear SMAD2/3 signaling | 2 | *DAXX SKI* | | C=301; O=2; E=0.11; R=18.24; rawP=0.0049; adjP=0.0458 | |
|  |  | TGF-beta receptor signaling | 2 | *DAXX SKI* | | C=301; O=2; E=0.11; R=18.24; rawP=0.0049; adjP=0.0458 | |
| **Table S1 continued** |  |  |  |  | |  | |
| **Log-ΔzFEV1_IP_** | PC | ALK1 signaling events | 2 | *DAXX SKI* | | C=317; O=2; E=0.12; R=17.32; rawP=0.0054; adjP=0.0458 | |
|  |  | Integrin-linked kinase signaling | 2 | *DAXX SKI* | | C=637; O=2; E=0.23; R=8.62; rawP=0.0206; adjP=0.0798 | |
|  |  | Class I PI3K signaling events mediated by Akt | 2 | *DAXX SKI* | | C=1254; O=2; E=0.46; R=4.38; rawP=0.0718; adjP=0.0798 | |
|  |  | Signaling events mediated by VEGFR1 and VEGFR2 | 2 | *DAXX SKI* | | C=1262; O=2; E=0.46; R=4.35; rawP=0.0726; adjP=0.0798 | |
|  |  | Syndecan-1-mediated signaling events | 2 | *DAXX SKI* | | C=1266; O=2; E=0.46; R=4.34; rawP=0.0730; adjP=0.0798 | |
| **ΔzFEV1/FVC_IP_** | GO | Identical protein binding | 3 | *MTHFD1L ANXA9 PRKRA* | | C=836; O=3; E=0.41; R=7.29; rawP=0.0059; adjP=0.0486 | |
|  |  | Protein dimerization activity | 3 | *MTHFD1L ANXA9 PRKRA* | | C=935; O=3; E=0.46; R=6.52; rawP=0.0081; adjP=0.0486 | |
| C: the number of reference genes in the category; O: the number of genes in the gene set and also in the category; | | | | | | |  |
| E: the expected number in the category; R: ratio of enrichment; rawP: p value from hypergeometric test; | | | | |  |  | |
| adjP: p-value adjusted by the multiple test adjustment (BH) | | |  |  | |  | |
